# Fabrication and Application of Zeolite/Acanthophora Spicifera Nanoporous Composite for Adsorption of Congo Red Dye from Wastewater

**DOI:** 10.3390/nano11092441

**Published:** 2021-09-19

**Authors:** Ahmed Hamd, Asmaa Ragab Dryaz, Mohamed Shaban, Hamad AlMohamadi, Khulood A. Abu Al-Ola, Nofal Khamis Soliman, Sayed A. Ahmed

**Affiliations:** 1 Nanophotonics and Applications Lab, Physics Department, Faculty of Science, Beni-Suef University, Beni-Suef 62514, Egypt; ahmed.hamd@nub.edu.eg; 2 Basic Science Department, Nahda University Beni-Suef, Beni-Suef 62764, Egypt; Nofal.Khamis@nub.edu.eg; 3 Chemistry Department, Faculty of Science, Beni-Suef University, Beni-Suef 62511, Egypt; drasmaaragab505@gmail.com (A.R.D.); skader_70@yahoo.com (S.A.A.); 4 Department of Physics, Faculty of Science, Islamic University in Madinah, Al-Madinah Al-Munawarah 42351, Saudi Arabia; 5 Department of Chemical Engineering, Faculty of Engineering, Islamic University of Madinah, Madinah 42351, Saudi Arabia; ham7171@gmail.com; 6 Department of Chemistry, College of Science, Taibah University, Al-Madinah Al-Munawarah 30002, Saudi Arabia; Kabualola@taibahu.edu.sa

**Keywords:** acanthophora spicifera, zeolite, nanocomposite, Congo red dye adsorption, wastewater treatment, kinetics and isotherms

## Abstract

Systematic investigations involving laboratory, analytical, and field trials were carried out to obtain the most efficient adsorbent for the removal of congo red (CR) dye from industrial effluent. Modification of the zeolite (Z) by the Acanthophora Spicifera algae (AS; marine algae) was evaluated in terms of adsorption capability of the zeolite to remove CR dye from aqueous solution. The zeolite/algae composite (ZAS) was fabricated using the wet impregnation technique. The AS, Z, and the synthesized ZAS composite were analyzed utilizing various characterization techniques. The newly synthesized ZAS composite has an adsorption capacity that is significantly higher than that of Z and AS, particularly at low CR concentrations. Batch experiments were carried out to explore the effects of different experimental factors, as well as the dye adsorption isotherms and kinetics. Owing to the presence of intermolecular interactions, the computational analysis showed that the adsorption of the CR molecule on zeolite surfaces is exothermic, energetically favorable, and spontaneous. Furthermore, growing the zeolite surface area has no discernible effect on the adsorption energies in all configurations. The ZAS composite may be used as a low-cost substitute adsorbent for the removal of anionic dyes from industrial wastewater at lower dye concentrations, according to the experimental results. Adsorption of CR dye onto Z, AS, and ZAS adsorbents was adequately explained by pseudo-second-order kinetics and the Langmuir isotherm. The sorption mechanism was also evaluated using Weber’s intra-particle diffusion module. Finally, field testing revealed that the newly synthesized adsorbent was 98.0% efficient at extracting dyes from industrial wastewater, proving the foundation of modern eco-friendly materials that aid in the reuse of industrial wastewater.

## 1. Introduction

Water is the planet’s primary source of life, as humans are well aware. Although industrialization and innovation improved humanity’s way of life, they also discussed the fundamental motivation to pollute clean water resources [1]. Heavy metals, dyes, pharmaceuticals, surfactants, pesticides, personal care items, and other chemicals are not only daily pollutant sources for unpolluted and limited water supplies, but they also harm all living beings [2,3,4,5,6]. Synthetic dyes produced from several industries, such as papers, rubbers, textiles, pigments, cosmetics, plastics, and printing, leave behind a large number of pollutants in clean water resources [2,7]. Dyes are used in a wide range of industries to color products and enhance the status of the products [8,9]. This extraordinary expansion of utilizing colors prompts water contamination in the environment [10,11]. These dyes are toxic, stable, and highly biodegradable [2,12]. Dyes also cause mutagenesis, carcinogenesis, chromosomal breaks, and respiratory poisonousness [13]. Congo red (CR) is an anionic dye that was chosen as a model dye because its color is one of the first contaminants to be identified in wastewater, and the presence of even very minute levels of CR in water is highly recognizable and unacceptable because it prevents water from re-oxygenating. When a person is exposed to Congo red dye, for example, they will experience severe eye irritation, skin irritation, stomach pain, nausea, vomiting, and diarrhea [14]. Various physical, chemical, and biological dye removal procedures have been reported. More precisely, coagulation, reverse osmosis, membrane separation, electrochemical, dilution, filtration, flotation, softening, and reverse osmosis techniques were used for this suggestion [15,16,17]. In comparison to the other methods, adsorption is the most convenient since it is inexpensive, simple, requires less maintenance, is easy to handle, and creates smaller amounts of sediment [18,19,20,21,22,23,24,25,26]. Among the used adsorbents, zeolite is a natural adsorbent with porous architectures. In addition to the traditional application of zeolite to soften water, it is used in catalytic processes, membrane separation, alternative zeolite processing, wastewater treatment, antimicrobial applications, construction, coating, pulps, and paper [27,28,29,30,31,32]. However, its dye adsorption capability is restricted by its compacted structure (low pore volume), which reduces its cationic exchange capacity and adsorption capabilities. Due to the zeolite’s surface and the dye molecules’ negative charges, the adsorption of anionic dyes on natural zeolite is relatively limited [33]. Various agents have been used to modify the surface of zeolite in order to boost the adsorption capacity of anionic dyes from wastewater. Previously, many reports discussed its modification by different surfactants to improve its sorption properties [34]. However, surfactants used as modifying agents, on the other hand, are commonly synthetic long-chain molecules with limited biodegradability and potential toxicity in the environment.

In recent decades, biomass wastes, fly ash, algae, clay minerals, and agricultural leftovers have all were employed as low-cost and effective adsorbents for removing dye and heavy metals from wastewater [35,36,37,38]. Phenols, alkaloids, saponins, flavonoids, and steroids are phytochemical constituents of the Acanthophora Spicifera (AS) marine red alga, and they contain a variety of active functional groups such as carboxylic, hydroxyl, amino, carbonyl, phosphates, and sulfonic [39]. Pollutants could be attracted strongly to the wall of the biomaterials such as AS alga because they contain active functional groups (such as carboxylic, hydroxyl, amino, carbonyl, phosphates, and sulfonic) [40]. Therefore, producing bio-capped zeolite-based adsorbent for dye removal from wastewater is quite important.

In this work, zeolite/algae composite (ZAS) is fabricated using the wet impregnation technique and applied as a nanoadsorbent of CR dye. In order to identify the best-optimized strategy for removing dyes, particularly CR dye, from industrial wastewater, we conducted a comprehensive study that comprised some computational calculations, laboratory, and field studies. To investigate the effect of AS on the uptake capability of Z, the adsorption capacity of Z, AS, and ZAS composite to remove the Congo red dye from wastewater was investigated under various experimental conditions. AS and Z were chosen for a variety of factors, including their abundance of natural adsorbent and low cost. Furthermore, the regeneration and reuse costs of Z, AS, and ZAS are lower, which may play a significant role in making this a viable operation. Furthermore, reusing a low-cost adsorbent lowers the cost of residue removal. The effects of starting dye concentration, contact time, adsorbent dosage, temperature, pH, and metal adsorption isotherms and kinetics on dye removal were investigated using batch experiments.

## 2. Materials and Methods

### 2.1. Materials

The El-Nassr company provided zeolite ore for mining, which was used without further alteration. AS algae were collected from the intertidal zone of the Egyptian Red Sea shores. Sigma-Aldrich provided the Congo red dye, which was dissolved in distilled water. Sigma-Aldrich supplied sodium hydroxide granules with a purity of 99.99% and hydrochloric acid (36%) for pH adjustment (Sigma-Aldrich, Munich, Germany).

### 2.2. Preparation of Zeolite/Algae (ZAS) Composite

The zeolite/algae composite (ZAS) was fabricated using the wet impregnation technique [41,42]. The following steps were used to make the zeolite/algae composite: in the first step, 1 g of zeolite and 1 g of algae were mixed in 20 mL of distilled water and stirred on a magnetic stirrer at 500 rpm for 60 min, then in ultrasonic for 60 min and repeated three times, after which the zeolite/algae composite was filtered and washed several times with distilled water and dried in a vacuum oven at 60 °C for 24 h.

### 2.3. Preparation of the Adsorbate

Congo red (CR), a common anionic dye, was used as the adsorbate in this experiment. Congo red dye is the sodium salt of 3,30-([1,10-biphenyl]-4,40-diyl) bis (4-amino naphthalene-1-sulfonic acid) with a formula: C_32_H_22_N_6_Na_2_O_6_S_2_. The structure of the dye molecule is shown in Figure S1 (Supplementary Materials). A 1000 mg/L stock solution was made by dissolving the required amount of CR (1000 mg) in a liter of distilled water. Working solutions were made by diluting the stock solution with distilled water to achieve the desired concentration. The pH of the solutions was adjusted to 3, 5, 7, and 10 using either 0.1M HCl or 0.1M NaOH solutions. The pH values were measured by a pH meter.

### 2.4. Samples Characterizations

The Z, AS, and ZAS composites were characterized using X-ray diffraction (XRD), Scanning electron microscopy (SEM), Fourier transformer infrared (FTIR) spectrometer, and optical spectroscopy. The XRD measurements were performed using the PANalytical diffractometer (Empyrean) that used Cu(Kα) source with a wavelength λα = 0.154045 nm and operated at 40 kV, 35 mA, and scan step of 0.02° between 20° and 70°. The average crystallite sizes, Ds, of the prepared nano-adsorbents were obtained by Scherer formula, Ds = 0.94 λα/βw cosΦ; where βw and Φ are the corrected full width at half maximum and the diffraction angle [43]. Quanta FEG 250 microscope (Switzerland) was used to measure SEM micrographs. The dry KBr pellet method was used to measure FT-IR spectra by a Bruker VERTEX 70 FT-IR spectrophotometer.

### 2.5. Adsorption Studies

Four adsorption experiment sequences were performed on Z, AS, and ZAS adsorbents under diverse adsorption circumstances. Table S1 shows the adsorption parameters that were investigated: starting CR concentration, reaction temperature, adsorbent dosage, and starting pH value (Supplementary Materials). All CR adsorption investigations were conducted in batch mode with continuous shaking and varied adsorption parameters such as CR starting concentration (5–25 mg/L), adsorption period (up to 480 min), adsorbent dose (0.01–0.05 g per 20 mL of CR solution), pH (3–10), and temperature (25–90 °C). In each experiment, the CR solution volume was set at 20 mL. Tracking the absorption peak using a Perkin Elmer Lambda 950 UV/Vis/NIR spectrophotometer revealed the variation in CR concentration. The reusability tests of Z, AS, and ZAS adsorbents were tested for four cycles at 25 °C and pH7 with 20 mg of each, 20 mL of 10 mg/L starting CR concentration, and 480 min reaction time. After each cycle; the adsorbent was removed from the solution, rinsed with distilled H_2_O, and set for the next cycle. Equations (1) and (2) were used to calculate the CR uptake amounts at equilibrium (q_e_) and after a period t (q_t_), as well as the CR removal% [44,45]:

(1) $$q_{i}=\left(C_{o}-C_{i}\right)\frac{V}{m};\;i=e,\;t$$

(2) $$\mathrm{CR}\;\mathrm{dye}\;\mathrm{removal}\;\%=\frac{{\left(C_{o}-C_{t}\right)}_{}}{C_{o}}\times 100$$

at which C_o_ is the starting CR concentration in mg/L, and C_i_ is the concentration of CR after period t (i = t) and at equilibrium (i = e). V and m are the CR volume in milliliters, and the ZAS mass in milligrams, respectively. The data points shown are the averages of three different trials.

### 2.6. Adsorption Isotherms

Langmuir, Freundlich, and Tempkin models were used to explain the reaction isotherms of the designed Z, AS, and ZAS nanocomposite for the examined CR [46,47,48]. All linear isotherms equations and their parameters are explained in the Supplementary Materials. The value of the dimensionless separation factor (R_L_) based on Equation (3) could be used to predict the degree of favorability of the Langmuir isotherm for equilibrium data [49].

(3) $$R_{L}=\frac{1}{\left(1+K_{L}C_{\max}\right)}$$

where C_max_ denotes the maximum initial CR concentration, and K_L_ represents the Langmuir constant. The K_L_ is a measure of the adsorbate ion affinity to the adsorption sites. Strong adsorption is indicated by a higher K_L_ value, whereas weak adsorption interaction is indicated by a lower K_L_ value. Unfavorable sorption is denoted by a value of R_L_ > 1, linear sorption by a value of R_L_ = 1, favorable sorption by a value of 0 < R_L_ < 1, and irreversible sorption by a value of R_L_ = 0. R_L_ > 1 refers to unfavorable sorption, R_L_ = 1 refers to linear sorption, 0 < R_L_ < 1 refers to favorable sorption, and R_L_ = 0 refers to irreversible sorption process.

### 2.7. Adsorption Kinetics and Mechanism

Several adsorption processes and kinetics models, including intra-particle diffusion, pseudo-first-and second-order models, and the Elovich kinetic model, are examined to determine the adsorption mechanisms and kinetics that fit the CR adsorption onto Z, AS, and ZAS adsorbents [4,50,51,52,53,54,55]. All linear kinetics equations and their parameters are explained in Supplementary Materials. The average values of all adsorption findings were measured in triplicates. The values of regression coefficients (R^2^) for various kinetic and isotherm models were obtained using OriginPro 2018’s statistical functions.

### 2.8. Computational Calculations

For the optimization of clinoptilolite and CR structures using density functional theory (DFT), the Generalized Gradient Approximation—Perdew–Burke–Ernzerhof (GGA-PBE) function was chosen. The method of choice was the dual numerical basis with polarisation (DNP). The spin-polarization effects were ignored when modeling the exchange/correlation function. Effective core potentials approximation was used to manage the core electrons of the clinoptilolite and CR structures. The DMol3 module of the Biovia Materials Studio was used to perform these calculations [56,57,58]. This investigation generated the original structure of zeolite clinoptilolite [59]. Zeolite unit cell bulk structure was subjected to energy minimization followed by simple box nanoclusters creation with sizes 7, 5 and 3 nm using a builder inherent in the studio software, as follows: simple box, 70 Å (X, Y, Z directions = 70 Å, K = 684, Na = 6112, Si = 468, O = 8834, Ca = 644, Al = 3214, H = 2720 atoms), 50 Å (X, Y, Z directions = 50 Å, K = 249, Na = 492, Si = 2213, O = 5520, Ca = 270, Al = 1342, H = 1044 atoms) and 30 Å (X, Y, Z directions = 30 Å, K = 64, Na = 132, Si = 468, O = 1220, Ca = 56, Al = 522, H = 224 atoms). The Monte Carlo (MC) simulation software was used to investigate the effect of varying clinoptilolite sizes on the energy of adsorption and to precisely place the active desorption sites of CR on the clinoptilolite surface. The Adsorption Locator module in Materials Studio was used to run the MC simulations, with the COMPASS force field as the force field and the current as the charge sector. The essential ideas of MC simulation were developed by Frenkel and Smit [60].

### 2.9. Field Experiments

The newly synthesized nanocomposite was tested as an effective eco-friendly adsorbent that could be applied on a large scale to remove industrial waste dye from industrial wastewater. To this end, wastewater containing waste dye was obtained from a clothing dying plant in Beni-Suef city, and the wastewater containing waste dye was used as is, with no further treatment or dilution. Based on our modified computational and experimental findings, the best nanoadsorbent system was chosen.

## 3. Results and Discussion

### 3.1. Characterization of the Adsorbents

#### 3.1.1. Morphological Properties

**Figure 1:**
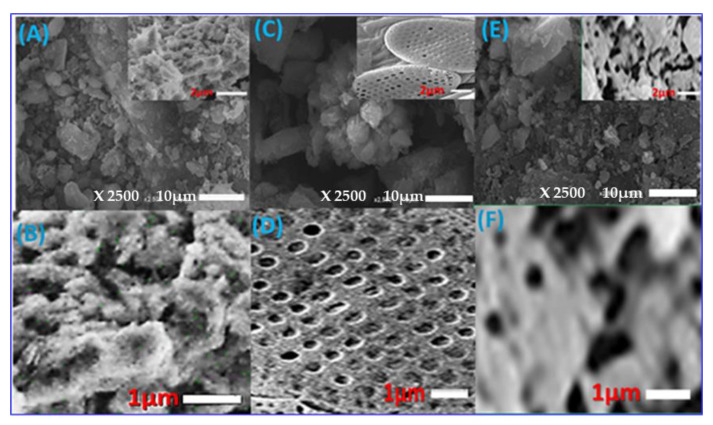
SEM micrographs of (**A**,**B**) Z, (**C**,**D**) AS, and (**E**,**F**) ZAS adsorbents.

The SEM images of Z, AS, and ZAS adsorbent are shown in Figure 1. The SEM images of zeolite (Figure 1a,b) show agglomerated rounded regular shape particles, rough surfaces, different particle sizes, and nanoporous cavities on the surface. The diameters of the nanopores ranged from 37.2 to 93.1 nm. Figure 1c,d shows that AS has a less rough surface with two sequences of nanopores, which impacts the surface area of AS and thus its adsorption capacity. The diameters of the large pore sequences range from 369.1 to 473.8 nm, while the diameters of the fine pore sequences range from 68.9 to 107.1 nm. When zeolite is treated with AS algae, the SEM images of the composite (Figure 1e,f) show that the pores in the AS surface are filled or covered with agglomerated zeolite particles. The diameters of the observed pores on the surface of the composite are 294.3 ± 56.7 nm. The formation of the ZAS composite could be confirmed by the observed changes in the morphological features of the composite as compared to that of Z and AS (Figure 1). The hydrodynamic diameter and particle size distribution of the Z, AS, and ZAS are estimated using the DLS technique (Supplementary Materials Figure S2). The average hydrodynamic diameters of Z, AS, and ZAS are ~80.4, 115.1, and 110.9 nm, respectively. Furthermore, the PET surface areas of Z, AS, and ZAS are ~91, 187, and 174 m^2^/g, respectively.

#### 3.1.2. X-ray Diffraction Characterization 

**Figure 2:**
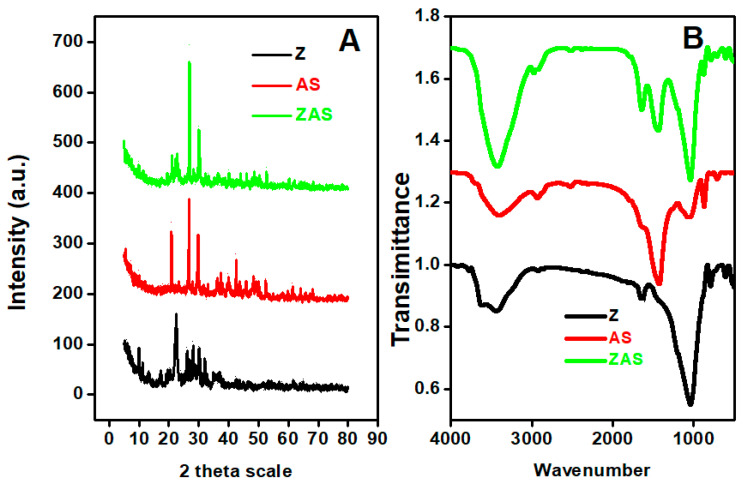
(**A**) XRD and (**B**) FTIR charts of Z, AS, and ZAS adsorbents.

XRD charts of Z, AS, and ZAS adsorbent are shown in Figure 2a. The characteristic diffraction peaks of zeolite mineral appear 2Φ of ~9.9°, 22.4°, 26.2°, 26.8°, 28.1°, 30.1°, and 32.0°, which are in line with other studies findings [61,62]. The major peaks of zeolite at 22.4° and 28.1° have d-spacing values of 3.97 Å and 3.17 Å, respectively. The XRD pattern of AS shows the characteristic main peaks at about 20.9°, 26.7°, 29.7°, 42.5°, 43.7°, 45.8°, 48.4°, and 42.5°. The major peaks of ZAS are seen in the XRD chart at around ~21.1°, 22.7°, 26.8°, and 30.0°. The Scherrer equation was used to calculate the average crystallite sizes for Z, AS, and ZAS, which were found to be 29.6, 92.7, and 92.8 nm, respectively, confirming their nanostructure nature.

The quantitative analysis of zeolite by XRF (X-ray fluorescence) shows that it consists of K_2_O with a weight% of 3.20%, CaO with 3.50%, Na_2_O with 0.78%_,_ SiO_2_ with 62.22%, MgO with 0.59%, Fe_2_O_3_ with 4.03%, TiO_2_ with 0.34%, ZrO_2_ with 0.11%, MnO with 0.12% and very small traces of Cl, BaO, ZnO, SrO, SO_3,_ and P_2_O_5_. For AS alga, flavonoids, alkaloids, steroids, saponins, and phenolic substances were all detected in preliminary qualitative tests. Thin-layer chromatography measurements revealed R_f_ values of 0.77 ± 0.2, 0.44 ± 0.01, 0.72 ± 0.01, 0.65 ± 0.01, 0.50 ± 0.02, respectively [34]. S.R. Sivakumar and K. Arunkumar’s method of flame photometric sodium and potassium detection, as well as sulphate colorimetric estimation at 470 nm, were used. This resulted in sulphate of 88.50 mg/g and Na^+^/K^+^ of 0.52 [63].

#### 3.1.3. FT-IR Analysis

FT-IR charts of Z, AS, and ZAS adsorbent are shown in Figure 2b. The spectrum showed the broadband of (OH) groups, where the bands at 3452, 3432, and 3442 cm^−1^ are characteristics of the inner OH stretching hydroxyl group [64,65]. In the case of zeolite, the band at 1029 cm^−1^ corresponds to the Si–O vibration mode, which is displaced to 1039 cm^−1^ for ZAS [66]. The 603 and 919 cm^−1^ bands are associated with Si–O–Al and octahedral aluminum (Al–OH), respectively [67]. The band at 464 cm^−1^ is associated with the Si–O–Si bending of zeolite, which is shifted to ~460 cm^−1^ for ZAS [67]. The bands that appeared in the region from 400 to 800 cm^−1^ are related to the metal oxides [68].

In the FT-IR spectrum of AS algae, the band at 3786 cm^−1^ is ascribed to the amine group (-NH) stretching, while the band at 3431 cm^−1^ is related to the hydroxyl group (-OH) of phenolic groups. The band at 2916 cm^−1^ was allocated to the alkyl groups (-CH) stretching, while the band at 1627 cm^−1^ is corresponding to the -C=O vibration. The mode at ~1426 cm^−1^ is ascribed to the C–H vibration [69]. The bands located around 1020 cm^−1^ refer to the sulfate group or the C–O bond [70]. Bands around 3300–3500 cm^−1^ are characteristic of the N–H stretching mode of amines. The mode at 2916 cm^−1^ refers to the O–H stretching mode of a carboxylic group [71]. In addition to the existence of AS bands, zeolite bands can be seen in the FT-IR spectrum of ZAS, indicating that zeolite was impregnated with AS to produce the ZAS composite. The band located at 2915 cm^−1^, stretching vibration of CH_2_ groups, in the ZAS composite (Figure 2b) suggests the interaction between alga function groups and active molecules on the surface of zeolite because it is not one of the characteristic bands of zeolite in the IR spectra. Absorption bands of ZAS composite were observed at around 3300–3600 and 1000–1660 cm^−1,^ and the intensities of the AS characteristic peaks notably increased with the incorporation of zeolite. Both band shift and band disappearance are in line with data obtained from other characterization techniques, which confirms the formation of the new ZAS composite. All bands shifts and assignments are summarized in Table S2.

### 3.2. Factors Influencing the Adsorption Process

#### 3.2.1. Influence of Starting CR Concentration

**Figure 3:**
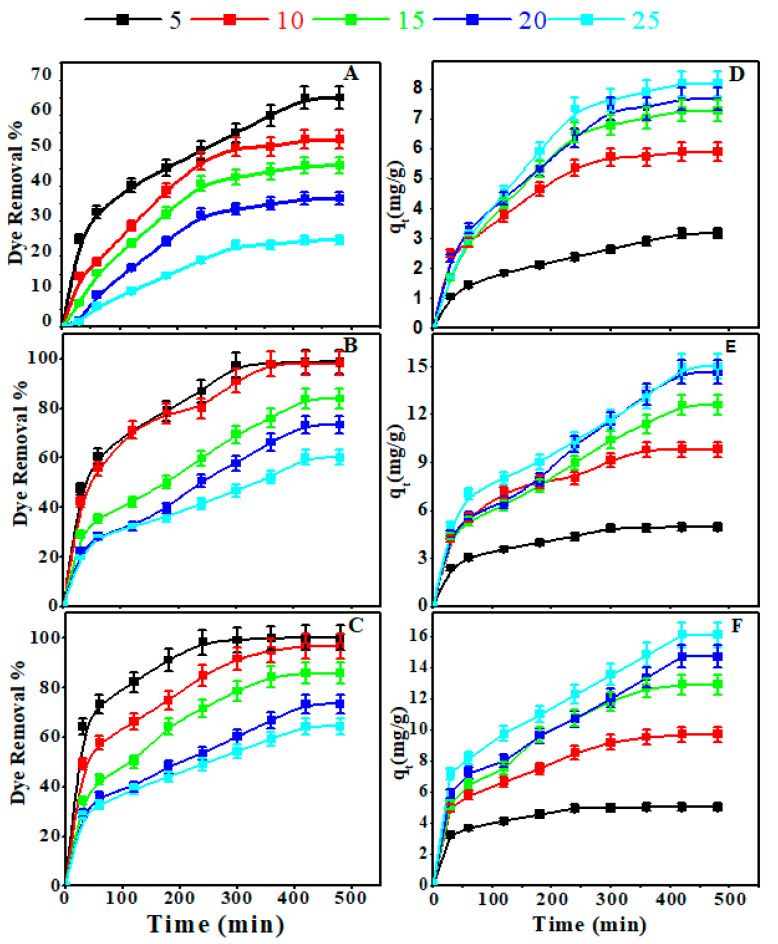
Effect of CR dye concentration and contact time on the removal% and the amount of CR dye adsorbed at 25 °C and pH 7 by 20 mg of (**A**,**D**) Z, (**B**,**E**) AS, and (**C**,**F**) ZAS.

The amount of CR removed by adsorption is highly dependent on the starting CR concentrations. The variations in the removal% and the amount of CR adsorbed using Z, AS, and ZAS adsorbents at different initial concentrations with time are illustrated in Figure 3a–f. The adsorption capacities and dye removal percentages are typically quite high during the initial stage of the adsorption process. Their growth is then slowed until the equilibrium state is reached. The presence of a large number of exposed active adsorption sites on the adsorbent surface may account for the high removal rate early in the reaction. The active sites become fully occupied by the CR molecules as the contact period between adsorbent and adsorbate increases. Because of the repulsive forces that exist between the adsorbed CR molecules on the adsorbent surfaces and those in the bulk liquid phase, the adsorption process is highly expected to follow the Langmuir mechanistic [72]. Hence, as the starting CR concentration increases, so does the proportion of CR eliminated. On the other hand, the amount of dye adsorbed by the adsorbent increases when the starting CR concentration rises, possibly due to increased mass transfer driving forces at higher starting CR concentrations. At all concentrations, the ZAS nanocomposite showed better performance for CR adsorption, and the CR elimination percent was in the sequence ZAS > AS > Z. The presence of phytochemical constituents such as phenols, alkaloids, saponins, flavonoids, and steroids, which contain several active functional groups such as carboxylic, hydroxyl, amino, carbonyl, phosphates, and sulfonic, may account for the higher removal percent of adsorbents containing AS species, as well as the high porosity and surface area of the AS and ZAS samples as seen in SEM images and PET measurements [39,40].

#### 3.2.2. Influence of Adsorbent Dosage

**Figure 4:**
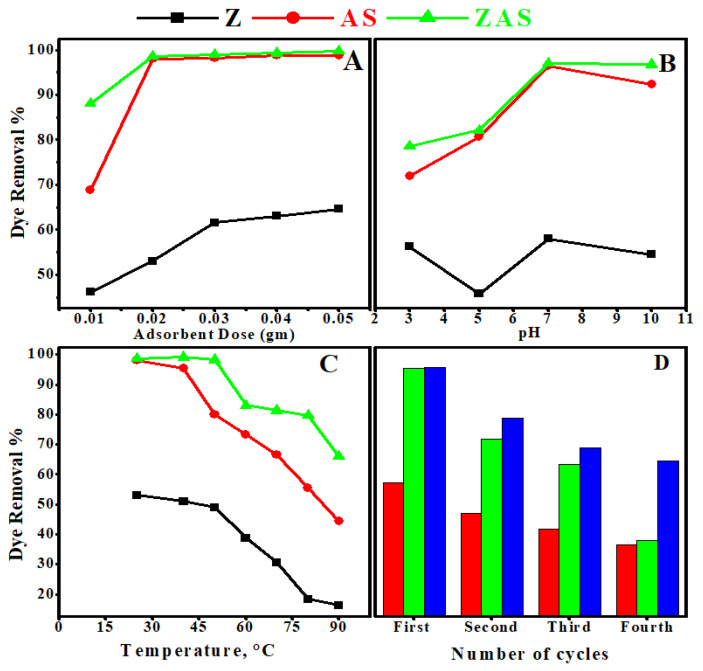
Effect of (**A**) adsorbent dose, (**B**) initial pH of the solution, (**C**) adsorption temperature, and (**D**) reusability test on the removal% of 20 mL CR solution of 10 mg/L by Z, AS, and ZAS.

The influence of adsorbent dose on CR removal percent is evaluated concerning adsorption cost at the optimal adsorbent dosage for best efficiency. Figure 4a depicts this graphically, with adsorbent dosages ranging from 0.01 to 0.05 g. Figure 4a shows that increasing the adsorbent dose from 0.01 to 0.05 g enhances the dye removal percent for all adsorbents; it rose from 46.2 to 64.6 percent for Z adsorbent, from 68.9 to 98.8 percent for AS adsorbent, and from 88.1 to 99.8% percent for ZAS adsorbent. This observation could be attributed to an increase in the frequency of active sites caused by increasing the adsorbent bulk [3,4,72]. It was observed that a large jump in removal% takes place by increasing the adsorbent dose from 0.01 to 0.02 g in the case of ZAS and from 0.01 to 0.03 g in the case of Z. By increasing the adsorbent dose over 0.02 g and 0.03 g in the case of ZAS and Z, respectively, the change in the removal% became slightly small. This could be related to the adsorbent’s screen effect, which occurs at higher adsorbent dosages [73]. Dense layers arise on adsorbent surfaces as a result of the accumulation of adsorbent molecules and the reduction in the space between them. The active sites were hidden from CR molecules by the formed dense layers. Furthermore, Z and ZAS overlapping resulted in a competition between CR molecules for restricted available binding sites. Agglomeration or aggregation at higher Z and ZAS dosages lengthen the diffusion paths for CR adsorption, lowering the adsorption rate [51,74,75,76].

#### 3.2.3. Influence of pH

pH is regarded as one of the most important elements regulating adsorbent dye removal capability in wastewater. The pH of the solution influences adsorption efficacy because changes in pH alter the degree of ionization of the adsorptive molecules as well as the surface properties of the adsorbent. Its effect on the adsorbent’s CR removal effectiveness was investigated between pH 3 and pH 10, as shown in Figure 4b. For 20 mL of 10 mg/L CR solution with a Z dose of 0.02 g, the Z adsorbent displays removal percentages of ~51.02%, 38.78%, 53.06%, and 48.98% at pH values of 3, 5, 7, and 10, respectively. The AS adsorbent shows removal percentages 69.39, 79.54, 98.00, and 93.08%, while ZAS adsorbent represents removal percentages 77.08, 81.25, 98.61, and 98.33% at pH values of 3, 5, 7, and 10, respectively, at the same previously mentioned conditions. As shown in Figure 4b, when the pH of the solution is 7, the adsorption capacities of CR on Z, AS, and ZAS reached their maxima. This may be because the interaction between adsorbent and CR is more pronounced than the interaction with OH^-^ ions in the solution [77]. At very low pH values, the positive charge on the solution interface grows, and the Z, AS, and ZAS surfaces seem positively charged. However, due to protonation, CR in the solution tends to be neutral. This scenario results in a decrease in anionic CR adsorption, as shown at pH 5 [78]. At high pH values, on the other hand, the positive charge on the solution interface decreases, and CR becomes negatively charged with OH^-^ ions. As a result, positively charged Z, AS, and ZAS interact competitively with negatively charged CR dyes or OH- ions. As a result, when pH exceeds 7, CR adsorption decreases once more [79].

#### 3.2.4. Influence of Temperature

The effect of temperature is an important physicochemical factor, as it causes variation in the adsorption capacities of the adsorbents [80]. The adsorption tests were conducted at 25, 40, 50, 60, 70, 80, and 90 °C, and the results are shown in Figure 4c. Where a decrease in CR removal% occurs with increasing temperature. This may be owed to the desorption manners resulted from the breakdown of adsorption forces that were liable for the adsorption of dye molecules on the adsorbent surface [81], which might be due to damaging of active sites, the weakening of adsorptive forces between active binding sites of the adsorbent and the adsorbate species [3,82,83]. As a result, the optimal temperature for CR adsorption is between 25 °C and 45 °C, especially for ZAS adsorbents. The drop in CR removal percentage as temperature rises indicates that adsorption is an exothermic process.

#### 3.2.5. Reusability Test

Z, AS, and ZAS reusability for the elimination of CR was repeated for four cycles using the same adsorbent dosage (Figure 4d). The data illustrated that; the removal% of all used adsorbents varied throughout the four adsorption cycles. For Z adsorbent, the recorded dye removal% was 53.1% at the first cycle, 40.8% at the second cycle, 34.7% at the third cycle, and 28.6% at the fourth cycle. For AS adsorbent, the dye removal% decreased from 98.0% at the first cycle to 30.0% at the fourth cycle. For the ZAS nanoadsorbent, the dye removal% decreased from 98.6% at the first cycle to 61.7% at the fourth cycle. The agglomeration of CR molecules on the surfaces of Z, AS, and ZAS adsorbents conceal the adsorbent surfaces and pores from the dissolved CR molecules, resulting in a reduction in adsorption capacity [84].

### 3.3. Adsorption Isotherms

**Figure 5:**
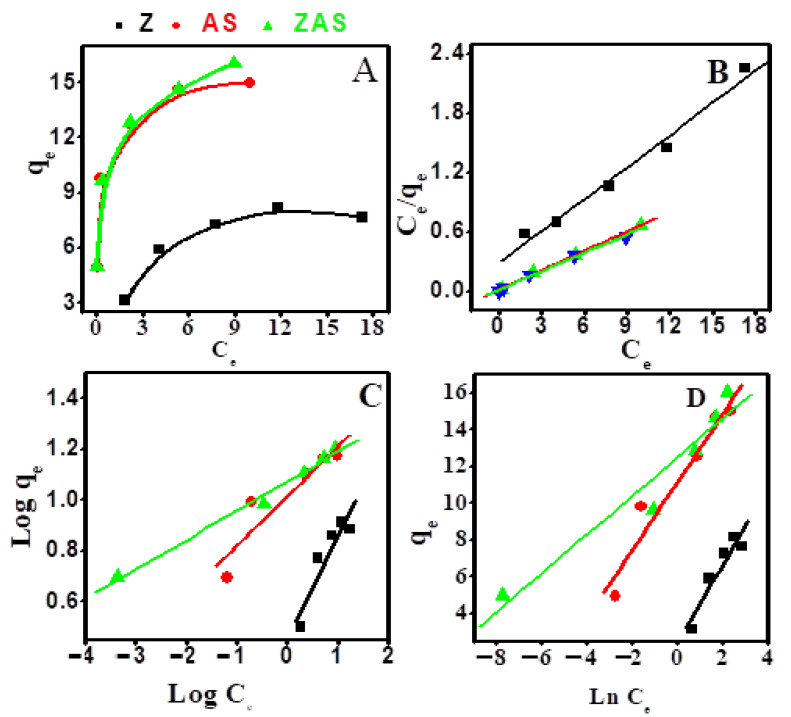
Plots of (**A**) Ce versus Qe, (**B**) Langmuir, (**C**) Freundlich, and (**D**) Temkin adsorption isotherms for the adsorption of CR dye by 20 mg of Z, AS, and ZAS at 25 °C and initial pH 7.

Figure 5a shows qe vs. Ce curves for Z, AS, and ZAs adsorbents to demonstrate the property of equilibrium adsorption. For the Langmuir, Freundlich, and Tempkin isotherms, these equilibrium curves were fitted. The statistical significance of correlation coefficient (R^2^) for linear fitting of Ce/qe vs. Ce, log(qe) vs. log(Ce), and qe vs. Ln(Ce) plots, respectively. The presented values of Q_o_, K_L_, K_F_, n, K_T_, B, and R^2^ in Table 1 are computed from the linear fitting of the plots in Figure 5b–d. The data in Table 1 reveal that CR adsorption on Z, AS, and ZAS adsorbents follow the Langmuir isotherm model with the best R^2^ value. Therefore, the elimination of the dye occurs at the active sites of the Z, AS, and ZAS adsorbents on a single surface layer (monolayer adsorption), and the adsorbed CR molecules do not react with each other. At 25 °C, the Langmuir isotherms of Z, AS, and ZAS have R^2^ values of 0.9795, 0.9985, and 0.9952, respectively. The value of R_L_ is less than one, indicating that CR adsorption in the studied case is valuable [85]. The maximum amount of CR adsorbed on the surface of Z, AS, and ZAS, according to the Langmuir isotherm model, was expected to be 9.25, 15.38, and 16.39 mg/g.

### 3.4. Adsorption Kinetics and Sorption Mechanism

**Figure 6:**
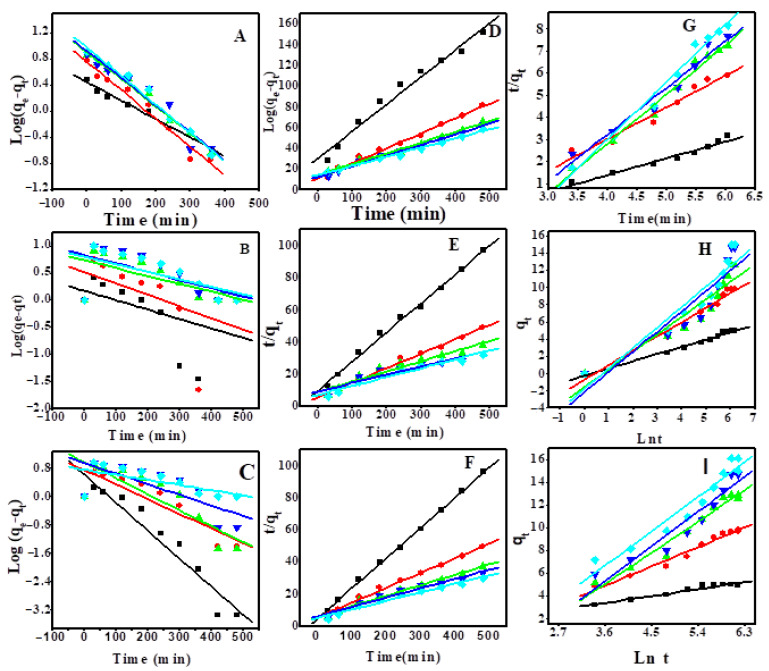
(**A**–**C**) pseudo-first-order, (**D**–**F**) pseudo-second-order, and (**G**–**I**) Elovich sorption kinetics of CR dye at 25 °C and pH 7 by 20 mg of Z, AS, and ZAS, respectively.

To study the most suitable kinetic model, the adsorption process of CR on Z, AS, and ZAS was followed at varying initial dye concentrations. Ln (q_e_–q_t_) vs. t, t/q_t_ vs. t, and q_t_ vs. ln(t) are used to illustrate the linear forms of the first-order, second-order, and Elovich kinetic models, Figure 6. The kinetic and statistical parameters, k_1_, k_2_, q_e_, β, α, and R^2^, are obtained and depicted in Table 2. The linear regression data in Table 2 show that CR adsorption onto Z, AS and ZAS is well fitted by second-order kinetics for the studied concentration range and models. At dye concentrations of 5, 10, 15, 20, and 25 ppm, R^2^ values for the second-order fit for ZAS are 0.9989, 0.9922, 0.9857, 0.9562, 0.9712, respectively. These R^2^ values are higher than those found for the Elovich and first-order models. Moreover, the behavior of AS adsorbent followed showed high R2 values, especially at low CR concentrations. This was further reinforced by the good approximation between the estimated q_e_ and experimental q_exp_. Two phases are involved in the pseudo-second-order adsorption mechanism. External diffusion refers to the transport of CR molecules from all sides to the outside surfaces of Z and AS. The CR molecules then adsorb and attach to the surfaces of Z, AS, and ZAS in a second stage.

**Figure 7:**
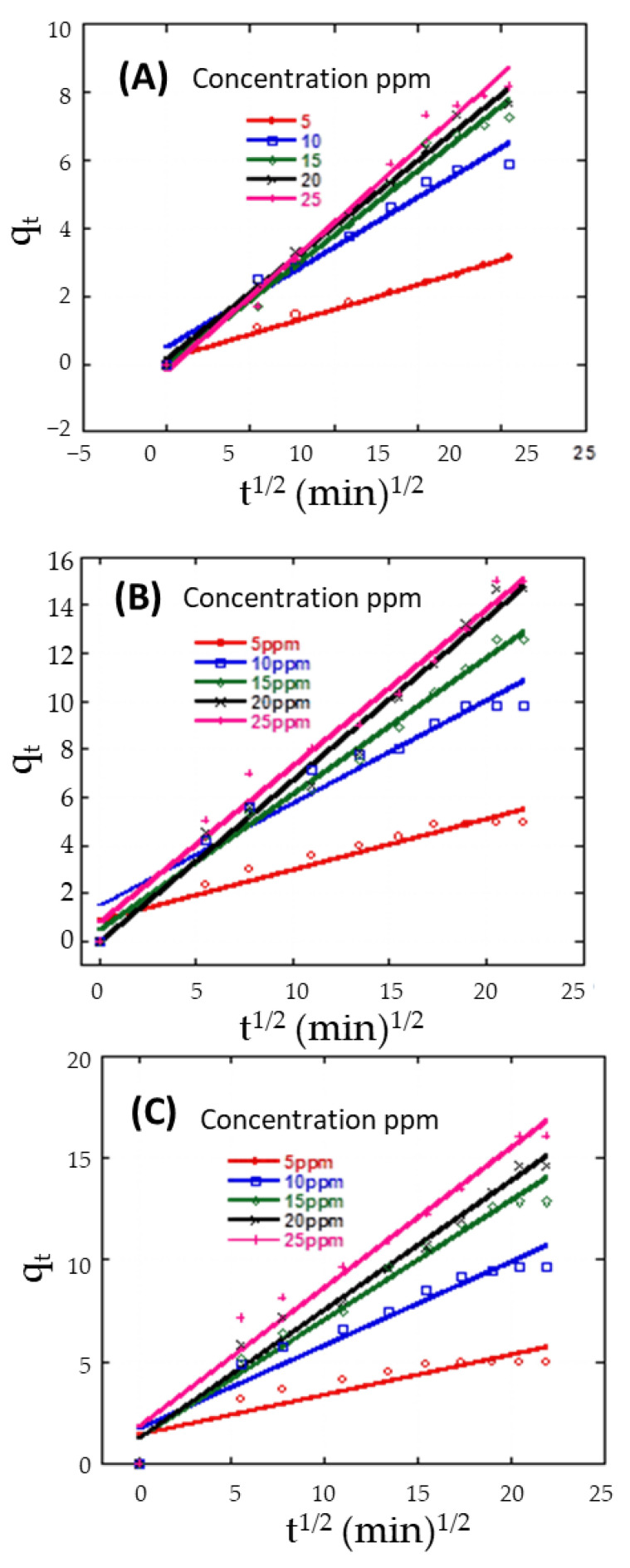
Intra-particle sorption kinetics of CR dye at 25 °C and pH 7 by 20 mg of (**A**) Z, (**B**) AS, and (**C**) ZAS.

To understand the mechanism and rate-controlling steps affecting adsorption kinetics. The experimental results were fitted for Weber’s intra-particle diffusion. A straight line in the chart of q_t_ versus t^1/2^ (Figure 7) proposes the applicability of the intra-particle diffusion model. K_3_ and I can be determined from the slope and intercept of the plot, respectively, and the results are provided in Table 2. The value of intercept I is not zero, demonstrating that the intra-particle diffusion model may not be the sole rate-controlling factor in determining the kinetics of the adsorption process [86]. The intercept in Figure 7 reflects the boundary layer effect. The larger the intercept, the greater the contribution of surface adsorption in the rate control step [86].

### 3.5. Computational Analysis

**Figure 8:**
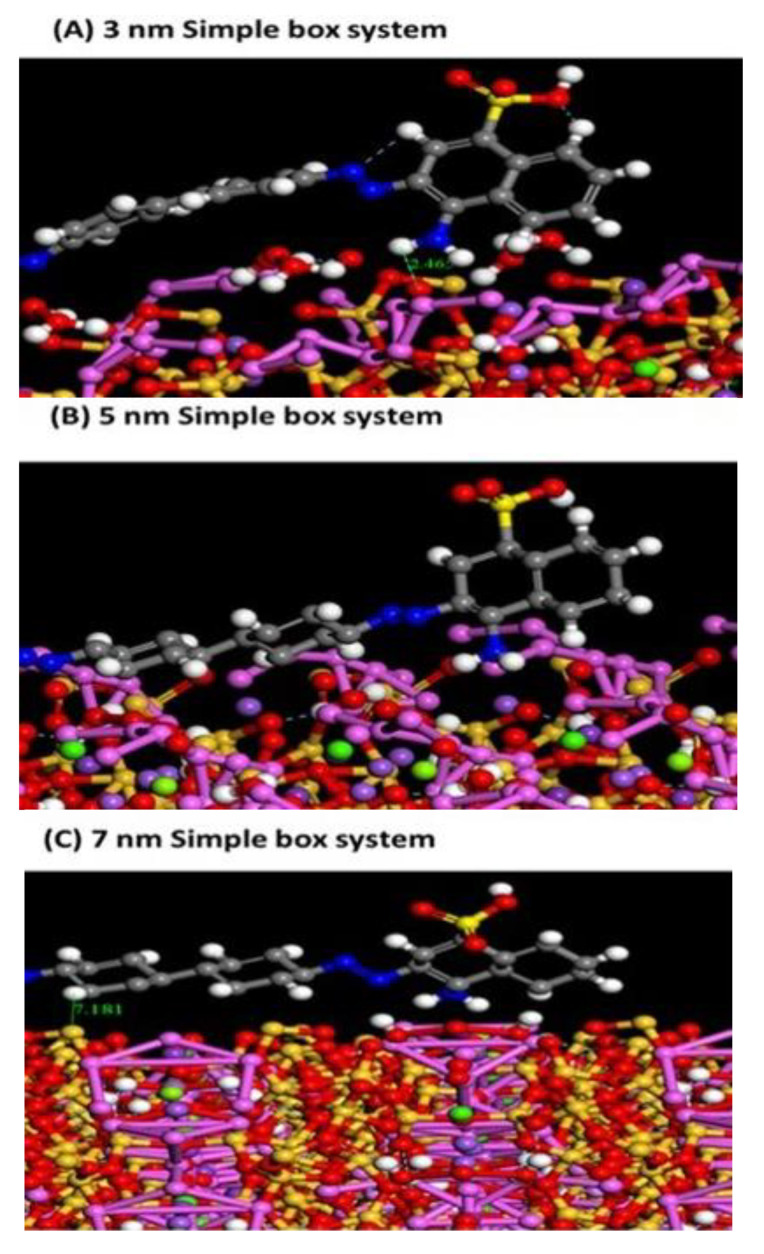
Snapshots for the adsorption configurations of CR/zeolite clinoptilolite of 3, 5, and 7 nm simple box systems, the bond length is in Angstroms.

Figure 8 reveals the minimum configurations obtained due to CR adsorption on clinoptilolite surface at three different sizes. The main target from MC simulation was to establish to what limit different planes and sizes of clinoptilolite will affect CR adsorption. Table 3 lists the adsorption energies of each zeolite clinoptilolite–Congo red system. Figure S3 (Supplementary Materials) shows the entire simple box system for adsorption configurations of Congo red on the clinoptilolite surface. Figure 8a–c illustrates simple snapshots of CR adsorption on the clinoptilolite’s surface (a dry system with no solvents containing 3, 5, and 7 nm simple box systems). Different H^-^ bond (HB) donor/acceptor regions were found clearly on CR molecules. Therefore, a number of HBs were formed between N and O atoms of the CR molecule and surface of clinoptilolite. Different possibilities were revealed in Figure 8, showing HBs and intramolecular HBs formed in a definite system (3 nm simple box); these bonds were lost in other systems (5 and 7 nm simple box). Table 3 summarizes the energy of adsorption (ΔE_ads_), energy of interaction (E_int_), and energy of deformation (E_def_) besides substrate–adsorbate configurations (dE_ads_/dN_i_), after removing one of the adsorbate components. Negative values of energy of adsorption for all configurations were obtained, indicating that the adsorption of the Congo red molecule on the surface of clinoptilolite is exothermic, energetically advantageous, and spontaneous due to the presence of intermolecular physical bindings. Moreover, increasing the size of the zeolite clinoptilolite has a considerable impact on the adsorption energy of all configurations, as adsorption energies decrease as the size of a simple box increases. Both HBs and intramolecular HBs between O and N of CR molecules and OH/H atoms of the clinoptilolite are weaker in the 5 and 7 nm simple boxes than that in the case of 3 nm simple box. This resulted in an obvious reduction in ΔE_ads_ values for 5 and 7 nm while larger values for 3 nm simple box systems, as shown in Table 3.

### 3.6. Field Experiment and Comparison with Other Adsorbents

Optimized parameters for the newly synthesized ZAS adsorbent including adsorbent weight (0.02 gm), near room temperature, the pH of the wastewater containing waste dye remained as it is without any changes, and contact time was 420 min. Scanning of wavelengths found in the as-received industrial wastewater revealed the presence of different wavelengths corresponding to different dyes. At the end of contact time, absorbance at different wavelengths was recorded to calculate the removal efficiency of the dyes from industrial wastewater. The data showed that the ZAS adsorbent extracted dyes from industrial wastewater with a 98.0% efficiency, validating the foundation of modern eco-friendly adsorbents that help with the reuse of industrial wastewater.

The adsorption capacities, q_m_, and removal percentages of different zeolite-based adsorbents reported in the literature were compared with that of Z, AS, and ZAS for adsorption of CR dye and are shown in Table 4. It shows that q_m_ values vary broadly for different adsorbents [87,88,89,90,91,92]. The results stated that Z, AS, and ZAS displayed a reasonable adsorption capacity for adsorption of CR dye from aqueous solutions in comparison with other adsorbents [87,88,89,90,91,92]. Moreover, according to the simple procedure that was used for the preparation of ZAS composite, availability, and price of both the Z and AS, the estimated cost of the preparation process of the ZAS composite is 1.5–2.0 USD/kg, which is very cheap if compared to the other adsorbents that reported in Table 4.

## 4. Conclusions

The hydrothermal treatment of Z and AS yielded a novel alga/zeolite composite (ZAS) which was used as a new adsorbent for CR from an aqueous solution. The experimental results demonstrated that decreasing the initial CR concentration increased the CR removal% and that the CR removal rate is high during the early stages of the adsorption experiment. The removal% increased by increasing Z, AS, and ZAS dosage from 0.01 g to 0.05 g. The temperature has a significant impact on the CR elimination percentage. The CR removal percent increased for all adsorbents as the initial pH of the CR solution was changed from 3 to 10, with the highest adsorption occurring at pH 7. The catalytic performances of Z, AS, and ZAS adsorbents were reduced to 28.6%, 30.0%, and 61.7% at the fourth cycle, respectively, in the reducibility test, indicating that the ZAS is more reusable as a catalyst than its components. The adsorption isotherm of CR onto Z, AS, and ZAS shows that Z, AS, and ZAS adsorbents track the Langmuir isotherm model. The CR adsorption onto Z, AS, and ZAS is well handled with the second-order model. The intra-particle diffusion kinetics model’s intercept is not zero, indicating that the intra-particle diffusion model may not be the main rate-controlling element in determining adsorption kinetics. Further studies are needed to look into the effects of dissolved salts on the adsorption behaviors of zeolite and ZAS composite, as well as the examination of more composites with varying ratios of the individual components.

## Figures and Tables

**Table 1:** Isotherm parameters for CR adsorption on Z, AS, and ZAS.

**Langmuir Isotherm**					
**Constant** **Adsorbent**	**Q_o_ (mg/g)**	**K_L_ (L/mg)**	**R_L_**		**R^2^**
**ZAS**	16.39	3.59	0.9951		0.011
**AS**	15.38	4.29	0.9984		0.009
**Z**	9.25	0.38	0.094		0.9794
**Freundlich Isotherm**					
**Constant** **Adsorbent**	**1/n**	**K_f_**		**R^2^**	
**ZAS**	0.116	11.40		0.9890	
**AS**	0.196	10.34		0.8601	
**Z**	0.404	2.85		0.8569	
**Temkin Isotherm**					
**Constant** **Adsorbent**	**B (J/mol)**	**K_T_ (L/mole)**		**R^2^**	
**ZAS**	1.0571	140,930		0.9890	
**AS**	1.856	416.5		0.8601	
**Z**	2.1487	2.85		0.8569	

**Table 2:** Parameters of the kinetic models for CR dye adsorption on Z, AS, and ZAS at 25 °C.

Adsorbent	Conc, Ppm	First-Order				Second-Order			Elovich Kinetic Model			Intraparticle Diffusion Model		
		q_e_ exp	q_e calc._	k_1_	R^2^	q_e calc._	k_2_	R^2^	β (g/mg)	α (mg/min)	R^2^	I	k_3_ (mg/g.min ^1/2^)	R^2^
**Z**	5	3.10	2.75	0.0028	0.9603	3.82	0.00228	0.9751	1.3158	0.0844	0.9597	0.167	0.146	0.9909
	10	5.90	5.75	0.0043	0.9682	6.94	0.00189	0.9918	0.7143	0.2035	0.9575	0.510	0.293	0.9661
	15	7.25	7.95	0.0041	0.9845	9.70	0.00073	0.9917	0.4546	0.1375	0.9835	−0.033	0.381	0.9821
	20	7.60	8.50	0.0042	0.9352	9.80	0.00087	0.9854	0.4762	0.1765	0.9630	0.168	0.389	0.9898
	25	8.10	9.50	0.0043	0.9798	11.36	0.00055	0.9890	0.3846	0.1510	0.9835	−0.175	0.435	0.9805
**AS**	5	4.93	1.49	0.0039	0.2210	5.53	0.00329	0.9945	1.1976	0.6439	0.9830	0.893	0.212	0.9160
	10	9.80	3.27	0.0048	0.2790	11.13	0.00138	0.9920	0.6050	1.1394	0.9730	1.490	0.427	0.9370
	15	12.58	5.42	0.0033	0.3980	15.86	0.00044	0.9398	0.4936	0.9474	0.8730	0.467	0.567	0.9870
	20	14.65	6.79	0.0035	0.3660	19.68	0.00027	0.8960	0.4255	0.9534	0.8271	0.0002	0.673	0.9810
	25	15.00	6.31	0.0032	0.3310	18.13	0.00042	0.9362	0.4237	1.1779	0.8860	0.793	0.653	0.9800
**ZAS**	5	4.99	4.08	0.0182	0.9201	5.31	0.00738	0.9989	1.4201	2.2864	0.9680	1.471	0.195	0.7910
	10	9.66	5.56	0.0094	0.7940	10.85	0.00155	0.9922	0.5309	0.6954	0.9680	1.727	0.409	0.9220
	15	12.85	9.10	0.0104	0.7260	15.35	0.00071	0.9857	0.3248	0.4324	0.9630	1.275	0.581	0.9698
	20	14.66	8.67	0.0069	0.5395	16.79	0.00056	0.9562	0.3037	0.4656	0.9130	1.251	0.634	0.9750
	25	16.07	5.73	0.0034	0.3867	18.56	0.00059	0.9712	0.2943	0.6577	0.9300	1.865	0.683	0.9650

**Table 3:** Energy of adsorption for adsorption configurations of Congo red on the surface of clinoptilolite with 3, 5, and 7 nm simple box systems.

Systems	Adsorption Energy	Rigid Adsorption Energy	Deformation Energy	Congo Red: dE_ad_/dN_i_
**3 nm**	−41.22350801	−31.58960227	−8.63390575	−41.22350801
**5 nm**	−39.83882700	−28.74763343	−10.09119357	−39.83882700
**7 nm**	−37.15356471	−27.49577995	−15.65778476	−37.15356471

**Table 4:** Comparison of the optimized conditions, removal%, and adsorption capacity of different zeolite-based CR adsorbents relative to our Z, AS, and ZAS nanoadsorbents.

Adsorbent	Conditions	Adsorption Capacity (q_m_) (mg/g)	Removal%	Reference
**Zeolite**	Concentration: 200 mg/L Dosage: 10 g/100 mL pH: 3 Temperature: 25 °C	4.3	95%	[87]
**ZnO@Ze Composite**	Concentration: 100 mg/L Dose: 50 mg pH: 3 Temperature: 25 °C Time: 60 min	161.3	99.5%	[88]
**SMZ6**	Concentration: 30 mg/L Dose: 40 mg pH: 6 Temperature: 20–40 °C Time: 24 h	69.94	98.7%	[89]
**PVA/SA/ZSM-5 zeolite membrane**	Concentration: 10 ppm Dosage: 2.5 wt% pH: 3 Temperature: 30 °C Time: 130 min	5.33	99.3%	[90]
**Na-zeolite@chitosan nanoparticle**	Concentration: 800 ppm Dose: 0.1 g pH: 5 Temperature: 25 °C Time: 60 min	0.00428 mmol/g	98.019%	[91]
**Cu(II)-incorporated zeolite Y**	Concentration: 10 mg/L Dosage: 1.5 g/L pH: 7 At room temperature Time: 240 min	-	87.72%	[92]
**Z**	Contact time: 480 min adsorbent dose: 0.02 g Concentration: 20 mg/L pH: 7 Temp: 25 °C	8.17	65	This work
**AS**		15	98.68	
**ZAS**		16.07	99.9	

## Data Availability

Data can be available upon request from the authors.

## Supplementary Materials

The following are available online at https://www.mdpi.com/article/10.3390/nano11092441/s1, Table S1: Conditions of experimental adsorption tests; Figure S1: Structure of Congo red; Figure S2. DLS spectra of Z, AS, and ZAS adsorbents; Figure S3. The adsorption configurations of adsorbed CR on zeolite clinoptilolite of 3, 5, and 7 nm simple box systems for clarity purposes.
