# Supplementary material for: Fabrication and Application of Zeolite/Acanthophora Spicifera Nanoporous Composite for Adsorption of Congo Red Dye from Wastewater
Source: Nanomaterials (Basel). 2021 Sep 19;11(9):2441. doi: 10.3390/nano11092441 (PMC8464800; doi:10.3390/nano11092441)
Supplement: Supplementary file 1 [file nanomaterials-11-02441-s001.zip › nanomaterials-1299866-supplementary.pdf]

nanomaterials

MDPI

Supplementary Materials

Fabrication and Application of Zeolite/Acanthophora Spicifera Nanoporous Composite for Adsorption of Congo Red Dye from Wastewater

Ahmed Hamd[1,2], Asmaa Ragab Dryaz[3], Mohamed Shaban[1,4,*], Hamad AlMohamadi[5], Khulood A. Abu Al-Ola[6], Nofal Khamis Soliman[2] and Sayed A. Ahmed[3]

1 Nanophotonics and Applications (NPA) Lab, Physics Department, Faculty of Science, Beni-Suef University, Beni-Suef, 62514, Egypt
2 Basic Science Department, Nahda University Beni-Suef (NUB), Beni Suef, Egypt
3 Chemistry Department, Faculty of Science, Beni-Suef University, Beni-Suef 62511, Egypt;
4 Department of Physics, Faculty of Science, Islamic University in Madinah, Al-Madinah Al-Munawarah 42351, Saudi Arabia
5 Department of Chemical Engineering, Faculty of Engineering, Islamic University of Madinah, Madinah, Saudi Arabia
6 Department of Chemistry, College of Science, Taibah University, Al-Madinah Al-Munawarah 30002, Saudi Arabia
* Correspondence: mssfadel@aucegypt.edu

1. Adsorption isotherms

Langmuir, Freundlich, and Tempkin isotherms have been applied to explain the reaction isotherm of the designed Z, AS, and ZAS nanocomposite for the tested CR. The three models can be represented by equations 1, 2, and 3, respectively [1-4]:

$$\frac{C_e}{q_e} = \frac{1}{K_L Q_o} + \frac{C_e}{Q_o} \tag{1}$$

$$\log q_e = \log K_F + \frac{1}{n} \log C_e \tag{2}$$

$$q_e = \mathrm{B} \ln K_T + \mathrm{B} \ln C_e \tag{3}$$

Here, Q_o is the maximum amount of dye removed by Z, AS, and ZAS adsorbents (mg/g); K_L, K_F, and K_T indicate to Langmuir constant, Freundlich constant, and Tempkin binding constant, respectively. B(=RT/b) is a constant associated with the adsorbed heat, n is the adsorption density, T is the absolute temperature, and R is the universal gas constant.

1. Adsorption kinetics and mechanism

Different adsorption mechanisms and kinetics models such as intra-particle diffusion, pseudo-first-order,pseudo-second-order, and simple Elovich kinetic model are used for identifying the adsorption mechanisms and kinetics models that best match with the adsorption of CR onto Z, AS, and ZAS adsorbents.

Equations 4 to 7 are used to represent the pseudo-first-order, pseudo-second-order, simple Elovich kinetic, and Intra-particle diffusion models, respectively [5-11].

$$\ln (q_e - q_t) = \ln(q_e) - k_1 t \tag{4}$$

$$\frac{t}{q_t} = \frac{1}{k_2 q_e^2} + \frac{t}{q_e} \tag{5}$$

$$q_t = \frac{1}{\beta}\ln \alpha\beta + \frac{1}{\beta}\ln t \quad (6)$$

$$q_t = k_3 t^{\frac{1}{2}} + I \quad (7)$$

Where k_1, k_2, and $k_3$represent rate constants of the pseudo-first-order, pseudo-second-order, and Intra-particle propagation models. I refers to a constant related to the boundary thickness. α implies the adsorption rate at time = 0 min(mg/min) and β represents the extent of surface coverage (g/mg).

Table S1. Conditions of experimental adsorption tests.

Scheme .	Dye concentration, ppm	Z, AS and ZAS weight, g	Temperature, °C	pH value
**1**	5, 10, 15, 20 and 25	0.02	25	7
**2**	10	0.01, 0.02, 0.03, 0.04 and 0.05	25	7
**3**	10	0.02	40, 50, 60,70, 80 and 90	7
**4**	10	0.02	25	3, 4, 5, 7, 8 and 10

Table S2. Characteristic wavenumbers and function groups of FTIR bands for Z, AS, and ZAS adsorbents.

FT-IR peaks (cm^{-1})			Assignment	References
**Z**	**AS**	**ZAS**		
-	3787	3300 – 3500	amine group (-NH) stretching	[12]
3452, 3432, and 3442	3432	2915	hydroxyl group (-OH)	[13-15]
-	2915	1425	(-CH) group	[16, 17]
-	1627	1019	(-C=O) group	[18]
1029	-	1039	Si-O-Al	[19]
464	-	461	Si-O-Si bending	[20]
400 - 800	-	400 - 800	metal oxides	[21]

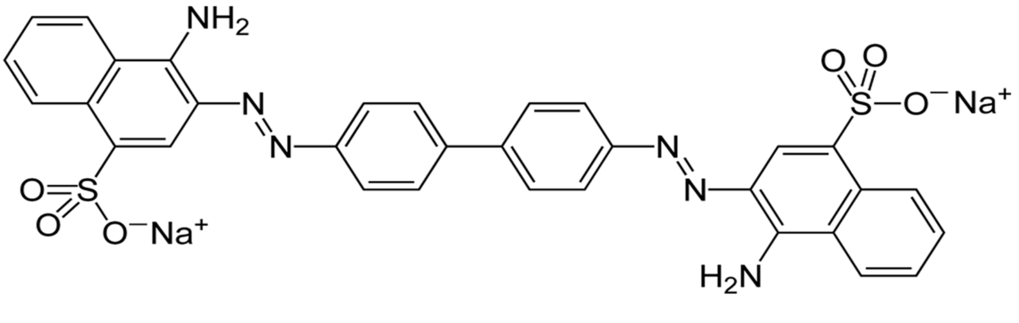

Figure S1. Structure of Congo red.

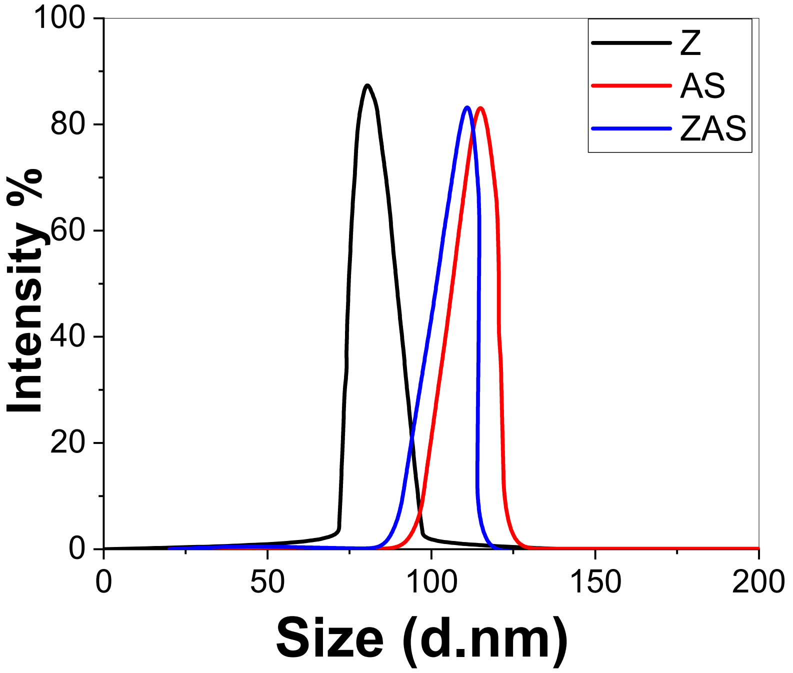

Figure S2. DLS spectra of Z, AS, and ZAS adsorbents.

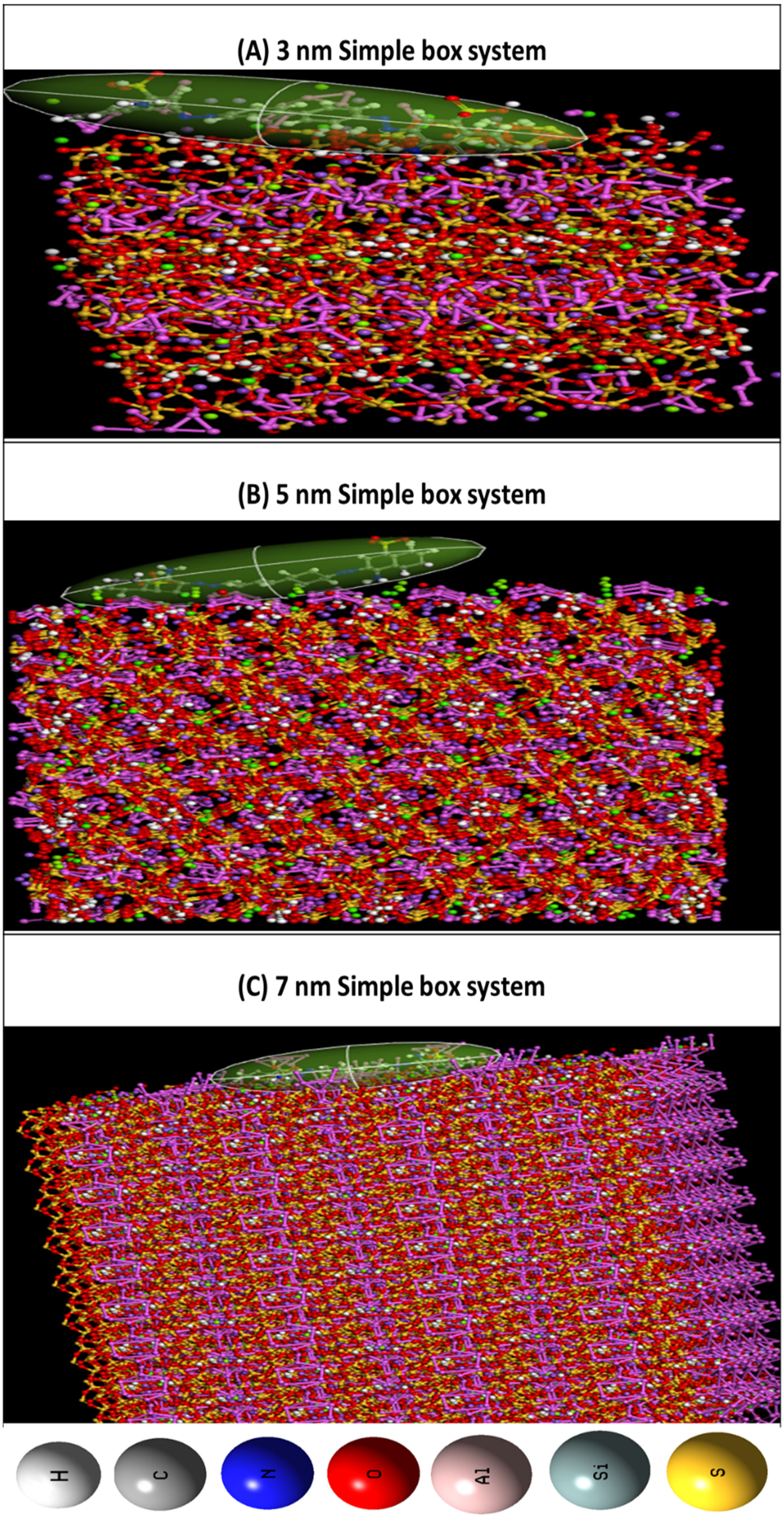

Figure S3. The adsorption configurations of adsorbed CR on zeolite clinoptilolite of 3, 5, and 7 nm simple box systems for clarity purposes.

References

1. Langmuir, I. THE ADSORPTION OF GASES ON PLANE SURFACES OF GLASS, MICA AND PLATINUM.. *J. Am. Chem. Soc.* **1918**, *40*, 1361–1403, https://doi.org/10.1021/ja02242a004.
2. Freundlich, H., Over the adsorption in solution. J. Phys. Chem, **1906**, 57, 1100–1107.
3. Foo, K.Y.; Hameed, B.H. Insights into the modeling of adsorption isotherm systems. *Chem. Eng. J.* **2010**, *156*, 2–10, https://doi.org/10.1016/j.cej.2009.09.013.
4. Temkin, M. and V. Pyzhev, Kinetics of ammonia synthesis on promoted iron catalysts. *Acta physicochim. URSS*, **1940**, 12, 217–222.
5. Soliman, N.; Mohamed, H.; Elsayed, R.; Elmedny, N.M.; Elghandour, A.H.; Ahmed, S.A. Removal of chromium and cadmium ions from aqueous solution using residue of Rumex dentatus L. plant waste. *DESALINATION Water Treat.* **2019**, *149*, 181–193, https://doi.org/10.5004/dwt.2019.23862.
6. Xin, N.; Gu, X.; Wu, H.; Hu, Y.; Yang, Z. Application of genetic algorithm-support vector regression (GA-SVR) for quantitative analysis of herbal medicines. *J. Chemom.* **2012**, *26*, 353–360, https://doi.org/10.1002/cem.2435.
7. Mohamed, H.; Soliman, N.; Abdelrheem, D.A.; Ramadan, A.A.; Elghandour, A.H.; Ahmed, S.A. Adsorption of Cd2+ and Cr3+ ions from aqueous solutions by using residue of Padina gymnospora waste as promising low-cost adsorbent. *Heliyon* **2019**, *5*, e01287, https://doi.org/10.1016/j.heliyon.2019.e01287.
8. Fan, L.; Luo, C.; Sun, M.; Qiu, H.; Li, X. Synthesis of magnetic β-cyclodextrin–chitosan/graphene oxide as nanoadsorbent and its application in dye adsorption and removal. *Colloids Surfaces B: Biointerfaces* **2013**, *103*, 601–607, https://doi.org/10.1016/j.colsurfb.2012.11.023.
9. Demiral, H.; Gündüzoğlu, G. Removal of nitrate from aqueous solutions by activated carbon prepared from sugar beet bagasse. *Bioresour. Technol.* **2010**, *101*, 1675–1680, https://doi.org/10.1016/j.biortech.2009.09.087.
10. Soliman, N.; Mohamed, H.; Ahmed, S.A.; Sayed, F.H.; Elghandour, A.H.; Ahmed, S.A. Cd2+ and Cu2+ removal by the waste of the marine brown macroalga Hydroclathrus clathratus. *Environ. Technol. Innov.* **2019**, *15*, 100365, https://doi.org/10.1016/j.eti.2019.100365.
11. Wu, F.-C.; Tseng, R.-L.; Juang, R.-S. Initial behavior of intraparticle diffusion model used in the description of adsorption kinetics. *Chem. Eng. J.* **2009**, *153*, 1–8, https://doi.org/10.1016/j.cej.2009.04.042.
12. Ibrahim, W.M.; Hassan, A.F.; Azab, Y.A. Biosorption of toxic heavy metals from aqueous solution by Ulva lactuca activated carbon. *Egypt. J. Basic Appl. Sci.* **2016**, *3*, 241–249, https://doi.org/10.1016/j.ejbas.2016.07.005.
13. Frost, R.L.; Horváth, E.; Makó, .; Kristóf, J. Modification of low- and high-defect kaolinite surfaces: implications for kaolinite mineral processing. *J. Colloid Interface Sci.* **2003**, *270*, 337–346, https://doi.org/10.1016/j.jcis.2003.10.034.
14. Pi, Z.; Liu, Z.; Yang, C.; Tian, X.; Fei, J.; Zheng, J. Exfoliation of kaolinite by urea-intercalation precursor and microwave irradiation assistance process. *Front. Earth Sci. China* **2007**, *1*, 26–29, https://doi.org/10.1007/s11707-007-0004-7.
15. Vaculikova, L., et al., Characterization and differentiation of kaolinites from selected Czech deposits using infrared spectroscopy and differential thermal analysis. Acta Geodyn. Geomater., **2011**, 8, 59–67.
16. Nautiyal, P.; Subramanian, K.; Dastidar, M. Adsorptive removal of dye using biochar derived from residual algae after in-situ transesterification: Alternate use of waste of biodiesel industry. *J. Environ. Manag.* **2016**, *182*, 187–197, https://doi.org/10.1016/j.jenvman.2016.07.063.
17. Ruthiraan, M.; Abdullah, E.; Mubarak, N.; Noraini, M. A promising route of magnetic based materials for removal of cadmium and methylene blue from waste water. *J. Environ. Chem. Eng.* **2017**, *5*, 1447–1455, https://doi.org/10.1016/j.jece.2017.02.038.
18. 18. Ghoneim, M.M.; El-Desoky, H.S.; El-Moselhy, K.M.; Amer, A.; El-Naga, E.H.A.; Mohamedein, L.; Al-Prol, A.E. Removal of cadmium from aqueous solution using marine green algae, Ulva lactuca. *Egypt. J. Aquat. Res.* **2014**, *40*, 235–242, https://doi.org/10.1016/j.ejar.2014.08.005.
19. Horváth, E.; Kristóf, J.; Frost, R.L. Vibrational Spectroscopy of Intercalated Kaolinites. Part I.. *Appl. Spectrosc. Rev.* **2010**, *45*, 130–147, https://doi.org/10.1080/05704920903435862.
20. Madejova, J. Baseline Studies of the Clay Minerals Society Source Clays: Infrared Methods. *Clays Clay Miner.* **2001**, *49*, 410–432, https://doi.org/10.1346/ccmn.2001.0490508.
21. Masoudi, R.; Moghimi, H.; Azin, E.; Taheri, R.A. Adsorption of cadmium from aqueous solutions by novel Fe3O4- newly isolated Actinomucor sp. bio-nanoadsorbent: functional group study. *Artif. Cells, Nanomedicine, Biotechnol.* **2018**, *46*, S1092–S1101, https://doi.org/10.1080/21691401.2018.1533841.
